# Relationship between combination antiretroviral therapy regimens and diabetes mellitus-related comorbidities among HIV patients in Gaborone Botswana

**DOI:** 10.1186/s12889-018-5232-0

**Published:** 2018-04-10

**Authors:** Jose Gaby Tshikuka, Goabaone Rankgoane-Pono, Mgaywa Gilbert Mjungu Damas Magafu, Tiny Masupe, Mooketsi Molefi, Maurice Nsikungu-Kalukul, John Thato Tlhakanelo, Shimeles Genna Hamda, Vincent Setlhare

**Affiliations:** 1 Department of Family Medicine and Public Health, Faculty of Medicine, University of Botswana, Gaborone, Botswana; 2 Department of Health Sciences, National Pedagogic University, Kinshasa I, Democratic Republic of the Congo; 3 Department of Global Health, University of Washington, Seattle, USA

**Keywords:** Diabetes mellitus-related comorbidities, Combination antiretroviral therapy, HIV patients, Recipients’ underlying risk factors, Survival function

## Abstract

**Background:**

Combination antiretroviral therapy (cARTs) regiments are known to prolong the recipients’ life even though they are risk factors for diabetes mellitus-related comorbidities (DRCs). We sought to: (i) examine cART relationship with DRCs among patients attending HIV clinics in Gaborone, Botswana (which cART regimens are associated with shorter/longer time to the event), (ii) characterize patients’ underlying biomedical and demographic risk factors of DRC and identify the most important, (iii) investigate survival of patients on different cART regimens in the presence of these risk factors.

**Methods:**

Data from two major HIV clinics in Botswana were reviewed. Relationships between different cART regimens and DRCs were investigated among 531 recipients. Recipients’ DRC risk factors were identified. Cox regression model was run. Unadjusted and adjusted hazard ratios were computed, and hazard and survival functions for different cART regimens were plotted.

**Results:**

Major findings were: patients on second- and third-line cART were less likely to develop DRCs earlier than those on first-line cART. Patients with CD4 count ≤ 200 cells/mm^3^ at cART initiation were more likely to develop DRCs earlier than those who had CD4 count > 200 cells/mm^3^. Overweight patients at cART initiation had a higher risk of developing DRCs earlier than those who had normal body mass index. Males had a lower risk of developing DRCs earlier than females.

**Conclusion:**

The risk of new onset of DRC among cART recipients is a function of the type of cART regimen, duration of exposure and patients’ underlying biomedical and demographic DRC risk factors. The study has provided a survival model highlighting DRCs’ significant prognostic factors to guide clinical care, policy and management of recipients of cARTs. Further studies in the same direction will likely improve the survival to the development of DRC of every cART recipient in this community.

## Background

Human Immunodeficiency Virus (HIV) patients are now living longer than before [1, 2]. This is mainly attributed to access to free combination antiretroviral therapy (cART) [2] or combinations consisting of a minimum of two active drugs from two classes of antiretroviral agents; usually they contain more different active drugs and are referred to as HAART. The improvement in life expectancy and quality of life observed among cART recipients could have been even better had these drugs not been associated with illnesses such as diabetes and diabetes-related comorbidities (DRC) [3, 4] or any comorbidity associated with Type 2 diabetes [5].

Association between cART and DRCs has been long suspected [4, 6, 7]. Studies have provided evidence showing that this association may depend not only on the duration of exposure to cART [8] but also the type of cART regimen taken [4]. In other words, while cART is considered a providential life-saving treatment for persons living with HIV (PLWH), it also has the potential of taking away lives by exposing recipients to an array of unwanted illnesses like diabetes mellitus and related complications [3]. This is a disturbing situation that makes the task of controlling and preventing morbidity and mortality among HIV patients more difficult, especially in settings with a high prevalence of HIV like in Botswana [9].

The initiative to provide free cART in Botswana started in 2002 [9]. Recipients of cART have access to standard treatment as defined by the Botswana national HIV & AIDS treatment guidelines [10] and the handbook of the Botswana integrated HIV clinical care guidelines [11] in use between 2002 and 2015. Components of cART regimens in Botswana were as presented in Appendix and described by Rankgoane-Pono and colleagues’ [12]. All these regimens have been to some extent, in different settings, associated with the development of DRCs after some time of exposure [6, 7, 13, 14]. Some experts have reported these associations only with regimens containing protease inhibitors [13], while others have described DRCs association with all types cART regimens [6, 7, 14]. The average time duration from cART initiation to the development of DRCs also significantly differed from one study to another [8, 14]. However, these results are perhaps due to differences in patient characteristics. For example, the patients’ underlying biomedical and demographic risk factors of DRC, such as being overweight or having CD4 cell count below 200 cells/mm^3^ have the potential to alter outcomes among patients if not well controlled [14]. Attributes associated with the risk of developing DRCs need to be identified earlier before patients’ enrolment in cART programs. This will allow for suitable care which minimizes cART adverse effects and delays or completely prevents DRC development. The availability of models highlighting DRCs’ significant prognostic factors is important to guide clinical care, policy and management of recipients of cARTs in a setting.

The study aimed to: (i) examine cART relationship with DRCs among patients attending HIV clinics in Gaborone, Botswana (which cART regimens are associated with shorter/longer time to the event), (ii) characterize patients’ underlying biomedical and demographic risk factors of DRC and identify the most important (those that significantly affect time to the event), (iii) investigate survival of patients on different cART regimens in the presence of these risk factors.

## Methods

### Study design

The study was a 12-year open retrospective cohort of cART recipients. The outcome event was DRC. This was recorded as diagnosed by the patient’s physician and listed in the 10th revision of the International Statistical Classification of Diseases and Related Health Problems (ICD-10) [5]. Exposures of interest were cART regimens and patients’ underlying biomedical and demographic characteristics. Survival was calculated as the time elapsed from the date of cART initiation until the date of DRC development. The study was conducted in Gaborone, Botswana’s capital city [15]. HIV period prevalence (2008–2013) was estimated at 19% among persons aged 18 months and above [16].Two clinics, Princess Marina Hospital (PMH) HIV clinic and Bontleng HIV clinic, were chosen as study sites out of 14 sites. The two clinics were selected because of their high capacity to cater for HIV patients and were the best at keeping patient records.

### Data collection

Data were collected from patient medical records. The records included patient files, referral and discharge booklets. Only data from patients who met the inclusion criteria were considered. A cohort that included: (i) patients who were receiving the first-line cART, (ii) patients who were receiving the second-line cART and (iii) those on the third-line cART was identified and followed between 2002 and 2015. The follow up endpoint was when DRC occurred or the end of the study. We excluded patients who had DRCs upon entry into the treatment program, pregnant women, recipients who were initiated on cART after 2012 (allowing for at least 3 years of follow-up), recipients aged less than 18 years and recipients with discrepant data from different records within the same clinic to assure data accuracies.

From each patient, we collected data on the date of enrolment into the program, the date of cART initiation, weight in kilograms (weight-1) and height in centimetres when entering the program, weight at the time of data collection (weight-2), CD4 cell count at cART initiation (CD4–1), CD4 cell count at the time of data collection (CD4–2), whether or not the treatment regimen was the same for a patient since the enrolment in the program, cART treatment regimen received before if there was a switch and reason for the switch. Only switches between different cART regimens were considered. Data on whether or not adherence to the treatment had been continued or discontinued and information on DRCs as well as the date of the diagnosis were also collected.

The sample size was estimated as by Hennekens and Buring [17], using a sampling error of 0.05 and a beta level of 0.20. The proportion of baseline DRC among HIV patients recipients of cART was 17.6% [18] and the expected magnitude of association between cART and DRC was set at 1.9 odds ratio. This led to an estimated sample size of 483. However, we included 540 participants in the study.

### Data analysis

Data were entered into a computer using Microsoft Excel (Redmond, WA) before being imported into IBM SPSS version 21 (Chicago, IL). Proportions of participants on the first-line cART, the second-line, the third-line and on second-, third-line cART regimens were estimated. Potential associations between the different cART regimens and DRCs were investigated by computing unadjusted hazard ratios (UHR) of each regimen and their 95% confidence intervals (CIs) using univariate Cox regression analysis.

Similarly, proportions of participants with underlying biomedical and demographic characteristics known as potential DRC risk factors were assessed, namely CD4 cell count as a continuous variable, CD4 cell count categories, gender, age as a continuous time dependent variable, age groups (< 35 years old/≥35 years old), body mass index (BMI) categories, whether patients adhered to treatment or not and attended PMH clinic or Bontleng clinic, and whether they switched cART regimens or not. Using DRCs as status /event/dependent variable, time in years, potential associations with these factors were assessed by computing UHR and their 95% CIs in univariate Cox regression analysis. Continuous variables namely CD4 cell count at cART initiation, CD4 cell count after cART initiation and age were not included in this analysis unless they were statistically significant in the two independent samples t-test analysis. Covariates that achieved *p* ≤ 0.9 in the univariate analysis were re-examined in a multivariate Cox regression model to identify those that significantly (*p* < 0.05) affected the time to event. Time spent in the study was expressed in years. Survival was estimated as the time elapsed from the date of initiation to cART until the date of the first DRC development or the last attendance. Recipients lost to follow-up, those referred to other facilities were left censored at the last date they were seen. Those who got to the end of the study without developing any type of DRC were right censored at the date of data collection. Adjusted hazard ratios (AHRs) and their 95% CIs were computed. The survival functions were plotted and the probability of survival in years from the date of initiation on: first-line cART (cART line1), second-line cART (cART line2), third-line cART (cART line3) or second-, third-line cART (cART line2/3) to the development of DRCs were compared using Log rank X^2^. The Cox regression presented herein is the best model where only covariates with *P* < 0.05 are kept in. The overall statistical significance of the model or how well the model fits the data was tested by computing the likelihood chi-square statistic.

### Ethical approval and consent to participate

Ethical approval to collect data from HIV clinics was sought and obtained from the University of Botswana Review Board and the ethics committee of the Ministry of Health and Wellness, Botswana. Permission to consult clinic record books and systems was also sought and obtained from the clinic management. As this was a record based study, no consent to participate was required.

## Results

Nine of the 540 records that were reviewed did not meet the inclusion criteria and were excluded. Thus, 531 patients’ records were included in the analysis. Of these, 368 (69.3%) were females. The mean age [± standard error of the mean (SEM)] of participants was 41.4 ± 8.9 years. The youngest was 19 years old while the oldest aged 82.0 years. The mean (± SEM) weight of patients at cART initiation was 60.6 ± 11.8 kg with the lowest record of 17.5 kg and 101.0 kg as the highest. After cART initiation the mean (± SEM) weight documented was 67.9 ± 14.50 kg, with the minimum of 32.5 kg and 117.7 kg as the maximum. The mean CD4 count (± SEM) at cART initiation was 139.5 ± 5.11 cells/mm^3^, the minimum count was 0.00 cells/mm^3^ and the maximum was 889.0 cells/mm^3^. After cART initiation, the mean CD4 count was 536.0 ± 10.16 cells/mm^3^ with the minimum count of 25.0 cells/mm^3^ and the maximum of 1441.0 cells/mm^3^. At cART initiation, 408 (76.6%) participants had a CD4 count of ≤200 cells /mm^3^compared to 34 (6.4%) after the initiation of cART. Four hundred and forty two patients or 83.2% did not develop any DRC, whereas 89 (16.8%) of them developed DRCs.

Results presented in Table 1 show proportions of participants by cART regimen and UHR as measures of crude association of each regimen with DRCs.

**Table 1:** Combination antiretroviral therapy (cART) regimen and unadjusted hazard ratio among recipients attending two HIV clinics in Gaborone, Botswana. Status variable: Diabetes mellitus-related comorbidities (DRCs) (*N* = 531)

	Proportion	Unadjusted		
Covariates	Number (%)	HR	95%CI	*P* value
On cART line1	318 (59.9)	2.03	1.26–3.27	0.004
On cART line2	209 (39.4)	0.48	0.30–0.79	0.003
On cART line3	4 (0.8)	0.95	0.13–6.83	0.90
On cART line2/3	213 (40.1)	0.49	0.31–0.79	0.004

HR = Hazard ratio, CI = confidence interval, On cART line1 = recipients of first-line combinations antiretroviral therapy, On cART line2 = recipients of second-line combinations antiretroviral therapy, On cART line3 = recipients of third-line combinations antiretroviral therapy, On cART line2/3 = aggregated recipients of second- and third- line combinations antiretroviral therapy

Patients on first-line cART were 2.03 times more at risk of developing DRCs earlier than those who were not on the first-line cART [UHR = 2.03; (95% CI: 1.26–3.27)]. Patients on second-line cART had a 52% less risk of developing DRCs earlier than patients who were not on the second-line cART [UHR = 0.48; (95% CI: 0.30–0.79)]. Those on third-line cART had 5% less risk of developing a DRC earlier than patients not on the third-line cART but the relationship was not statistically significant [UHR = 0.95; (95% CI: 0.13–6.83) and *p* = 0.9]. When patients on second-line cART were aggregated with those on third-line (cART line2/3), recipients showed 51% less risk of developing DRCs earlier than patients who were not on cART line2/3 [UHR 0.49, (95% CI 0.31–0.79).

Proportions of patients by biomedical and demographic characteristics and UHR as measures by their crude association with DRCs are shown in Table 2.

**Table 2:** Proportions of underlying biomedical and demographic characteristics and unadjusted hazard ratios among cART recipients attending two HIV clinics in Gaborone, Botswana. Status variable: Diabetes mellitus-related comorbidities (DRCs, *N* = 531)

Covariates	Proportion Number (%)	Unadjusted		
		HR	95%CI	P value
Male	163 (30.7)	0.58	0.38–0.89	0.012
Age ± SE	41.39 ± 0.38	1.03	1.01–1.05	0.006
≤35 years old	132 (24.9)	0.40	0.20–0.80	0.01
CD4–1 ≤ 200 cells/mm^3^	124 (23.4)	1.6	1.03–2.6	0.036
CD4–1 ≥ 350 cells/mm^3^	24 (4.5)	0.99	0.31–3.16	0–90
CD4–2 ≤ 200 cells/mm^3^	34 (6.4)	0.94	0.38–2.32	0.89
CD4–2 ≥ 350 cells/mm^3^	420 (79.1)	1,16	0.67–2.04	0.58
PMH Clinic	314 (50.1)	6.41	3.55–11.56	0.001
Adherence				
No	31 (5.8)	1	–	–
Yes	478 (90.0)	4.53	1.42–14.47	0.01
Unknown	22 (4.1)	1.14	0.41–3.12	0.79
^1^Switched cART Line				
Did not switch	235 (44.3)	1	–	–
From cART line1 to line 2	281 (52.9)	0.75	0.49–1.15	0.18
Unknown	15 (2.8)	1.28	0.46–3.58	0.63
BMI at cART				
Normal weight	337 (63.5)	1	–	–
Underweight	80 (15.1)	0.88	0.46–1.69	0.71
Overweight	114 (21.4)	1.63	1.02–2.61	0.043

HR = Hazard ratio, CI = confidence interval, ^1^Switched cART regimens = switched from the initial cART line to another line, Unknown = whether the patient switched regimen or not is unclear, BMI at cART = BMI at initiation on cART, CD4–1 cells/mm^3^ = CD4 count at initiation on cART, CD4–2 cells/mm^3^ = CD4 count after initiation on cART

UHR for CD4–1 and CD4–2 count as continuous variables are not shown here as both variables did not achieve significance in the exploratory two independent samples t-test analysis. Age as a continuous variable indicated that the hazard of developing DRCs increased by 3% [UHR = 1.03; (95% CI: 1.01–1.05)] for every one year increase in recipients age. Of all the characteristics investigated, seven showed significant crude associations with DRCs (*p* < 0.05) while others achieved *p* ≤ 0.9 and were considered for multivariate analysis, details on these results are given in Table 2.

Associations between cART regimens and DRCs after adjusting for biomedical and demographic factors of recipients are presented in Table 3.

**Table 3:** Combination antiretroviral therapy (cART) regimen and patient biomedical and demographic factors independently associated with the development of diabetes mellitus-related comorbidities (DRCs). Status or event variable^†^: Diabetes mellitus-related comorbidities (DRCs, *N* = 531)

Covariates	Proportion Number (%)	Unadjusted		Adjusted	
		HR	95%CI	HR	95%CI
cART Line1	0318 (59.9.1)	1	–	1	–
cART Line2/3	213 (40.1)	0.49*	0.31–0.79	0.53*	0.33–0.87
CD4–1 > 200 cells/mm^3^	407 (76.6)	1	–	1	–
CD4–1 ≤ 200 cells/mm^3^	124 (23.4)	1.6*	1.03–3.2	1.68*	1.03–2.73
Adherence					
No	31 (5.8)	1	–	1	–
Yes	478 (90.0)	4.53*	1.42–14.5	3.05©	0.92–10.0
Unknown	22 (4.1)	1.14	0.41–3.12	0.89	0.32–2.51
BMI at cART					
Normal Weight	337 (63.5)	1	–	1	–
Under weight	80 (15.1)	0.88	0.46–1.69	0.74	0.37–1.44
Overweight	114 (21.4)	1.6*	1.02–2.61	1.63*	1.07–2.64
Female	368 (69.3)	1	–	1	–
Male	163 (30.7)	0.58*	0.38–0.89	0.49*	0.32–0.77

^†^likelihood chi-square statistic = 36.25, *p* = 0.0001; CD4–1 = CD4 at initiation on cART, HR = Hazard ratio, CI = confidence interval, BMI at cART = BMI at initiation on cART, *P < 0.05, ©*p* = 0.06 (borderline); note that all variables, including age as continuous variable or age groups, that did not achieve *p* < 0.05 are not included in this model

HIV patients on second- and third-line cART (cART line2/3) were 47% less likely to develop DRCs earlier than those on first-line cART [AHR = 0.53; (95% CI: 0.33–0.87)]. Recipients of cART who had a CD4–1 count equal to or less than 200 cells/mm^3^ at cART initiation were 68% more likely to develop DRCs earlier than those who had a CD4–1 count greater than 200 cells/mm^3^ at initiation of cART [AHR = 1.68; (95% CI: 1.03–2.73)]. Participants who reported adherence to cART had a 3.1 times higher risk of developing DRCs earlier than those who did not adhere [AHR = 3.1; (95% CI: 0.92–10.0), *p* = 0.06]. Overweight patients at initiation of cART, had a 63% higher risk of developing DRCs earlier than recipients who had normal BMI at cART initiation [AHR = 1.63; (95% CI: 1.07–2.64)]. Males had a 51% lower hazard of the outcome than females [AHR = 0.49; (95% CI: 0.32–0.77)]. Age as a continuous variable did not enter the multivariate model as it did not achieve a *p* < 0.05.

**Fig. 1:**
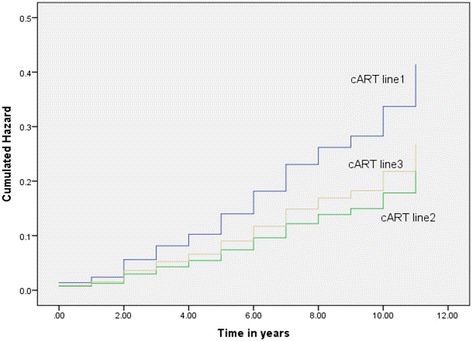
Hazard function for recipients of cART first-line, second-line and third-line: a 12 years retrospective cohort study in two HIV Clinics in Botswana. cART line1 = cART first-line regimen, cART line2 = cART second-line regimen, cART line3 = cART thrid-line regimen

The risk of new onset DRCs increased each year with cumulative exposure to each cART regimen under study**.** However, the most significant risk was documented among recipients of the first-line cART regimen while the least risk of new DRC cases per year of cumulative exposure to cART was recorded among recipients of the second-line cART regimen (Fig. 1).

**Fig. 2:**
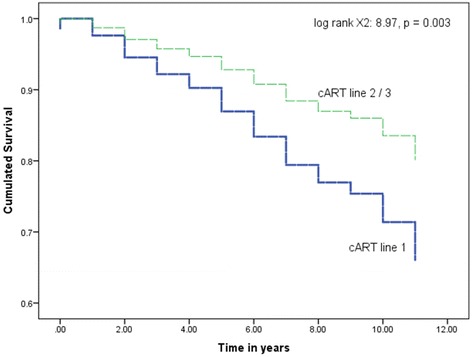
Survival function for recipients of cART first-line, and second-, third-line regimens: a 12 years retrospective cohort study in two HIV Clinics in Botswana. cART 2/3 = cART second-, and third-line regimen, cART line 1 = cART first-line regimen, log rankX^2^ = log rank chi square

The survival function of patients on second- and third-line regimens almost overlapped, with both functions disassociated with the first-line function. Data presented in Fig. 2 show survival functions of aggregated recipients of second- and third-line cART regimens in comparison with those of the first-line cART regimen.

The log rank (Mantel Cox) test confirmed that recipients of second-, third-line cART had significantly longer survival to DRC development than recipients of first-line cART (X^2^ = 8.97, *p* = 0.003).

## Discussion

Findings from this study bring new insights to the fight against HIV and related comorbidities. The risk of new DRC onset per year of cumulative exposure to cART was higher among recipients of first-line cART than in recipients of cART second-line and cART third-line regimens. The longest survival/time duration to the development of DRC per year of cumulative exposure to cART was documented among recipients of second- and third-line cART regimens. In other words recipients of the second-, and third-line cART were less likely to develop DRCs earlier than recipients of the first-line cART. This lends support to the claim that the survival of cART recipients to DRC development is not solely a function of the duration that patients are subjected to these drugs but also a function of the specific regimen they are exposed to [19, 20] and the recipients underlying biomedical and demographic characteristics [21]. While the overlapping of the second-, third-line regimen DRC hazard/survival functions may suggest comparable effects between the two cART regimens, they also indicate that recipients of both the second and third-line regimen had a longer survival probability compared to their counterparts on first-line regimen. This implies that had some patients been directly initiated on second- or third-line cART regimen, the outcome might have been much better for all the recipients.

In short, important findings in this study were that recipients of the second-, third-line cART regimen were less likely to develop DRCs earlier than first-line cART regimen recipients. This is good news that may indicate that different cART regimens are differently associated with DRCs and different groups of recipients. Thus, depending on circumstances, the current second- and third-line regimens may be considered for use as first-line regimen while taking into account factors such as recipients’ pre-existing DRC risk factors, drug availability, cost-effectiveness, etc. For instance, patients with characteristics such as overweight, female gender or CD4–1 equal or less than 200 cells/mm^3^ were identified in this study as at more risk of developing DRCs earlier than those without and should have been put directly on second- or third-line regimen rather than starting with the first-line treatment. Recipients aged 35 years or older also need particular attention compared to those aged less than 35 years before being assigned to a specific cART regimen.

However, the challenge lies in the cost difference between the first-line, the second- and third-line regimens and possible adverse effects of the third-line regimen. While WHO states that treatment programs using Tenofovir or AZT are promising, they may not be the ultimate solution to alleviate the situation since both drugs are known to be expensive [22] and yet to some extent associated with DRCs [6, 7, 23–25]. The fact that despite their high cost, these drugs are still associated to a certain degree with DRCs [23–25] calls for a meticulous drug selection that takes into account biomedical and demographic characteristics of recipients so as to improve their survival probability to the event.

As with any other study, this study had some limitations. It was a retrospective review of data and may be missing some important DRC risk factors like physical activity, cigarette smoking, alcohol consumption and family history of DRCs. Prospective studies are needed to address this particular issue. Even so, the study has provided useful evidence to inform policy and decision making on alternative approaches for the management and care of HIV patients. The study has also indicated that patients with underlying DRC risk factors such as overweight, female gender, aged 35 years or older or those with CD4 ≤ 200 cells/mm^3^ should be selectively assigned to treatment regimens for possible improvement of their survival.

## Conclusion

The study has shown that the risk of new DRC onset among cART recipients is not only a function of the type of cART regimen or the duration of exposure to these drugs but also a function of patients’ underlying biomedical and demographic DRC risk factors. The study has also presented a survival model highlighting DRCs’ significant prognostic factors to guide clinical care, policy and management of recipients of cARTs. Further studies in the same direction will likely improve the survival to the development of DRC of every cART recipient in this community.

## Appendix

**Table 4:** Standard First and Second Line ART Regimens in Botswana: Composition of cART lines

First line	First line Modifications	Second line	Second line Modifications
AZT +3TC (CBV-combivir) + EFV AZT + 3TC + NVP AZT + DDI+ EFV AZT+ DDI + NVP	TDF renal toxicity w/o CVD risk: ABC/3TC/DTG (If CVD rash: Consult HIV specialist) CNS Toxicity and/or Hepatic Toxicity: TRU/DTG	TDF + FTC + ALU	AZT Anemia and/or TDF Renal Toxicity: ABC/3TC/DTG
TDF+ FTC (or 3TC) + EFV TDF+ FTC (or 3TC) + NVP		CBV + ALU	If anemic ABC+ 3TC+ ALU
D4T + 3TC + EFV D4T + 3TC + NVP DDI +3TC + EFV DDI+ 3TC + NVP		TDF + FTC = ALU	If renal insufficiency but no anemia: CBV + ALU If renal insufficiency and anemia: ABC + 3TC + ALU

Third line regimen was made up of other alternative combinations or salvage therapy. This was deployed in case of failure of both the standard first and second-line regimens

The Botswana National ART programme also called Masa was launched in 2002. Over the years the Ministry of health and wellness has issued and updated ARV treatment guidelines based on WHO recommendations. First line and second line regimens have therefore been modified over the years from 2002 to 2016. Between 2002 and 2015, the period for this study, the treatment regimens were as presented in Appendix adapted from the 2012 and before 2016 treatment guidelines [9–12]

## Abbreviations

AHR: Adjusted hazard ratio
AIDS: Acquired Immune deficiency Syndrome
AZT: Azidothymidine, also known as zidovudine
BMI: Body mass index
Bontleng HIV clinic: one of the study site
cART: Combination Antiretroviral Therapy
CD4: Cluster of differentiation 4, glycoprotein found on the surface of T helper cells
CD4–1: CD4 Count at cART initiation
CD4–2: CD4 Count at the time of data collection
CI: Confidence Interval
DRCs: Diabetes Mellitus-Related Comorbidities
HIV: Human Immunodeficiency Virus
ICD-10: The 10th revision of the International Statistical Classification of Diseases and Related Health Problems, a medical classification list by the World Health Organization
Log-rank X^2^: Log-rank Chi-square
PLHIV: People Living with HIV
PMH HIV Clinic: Princess Marina Hospital HIV Clinic or another study site
SE: Standard Error
SEM: Standard Error of the Mean
Survival: Survival was calculated as the time elapsed from the date of cART initiation to the date of the development of the first DRC at the time of data collection
Time to the event: Time duration from initiation on cART to the occurrence of DRCs
UHR: Unadjusted hazard ratio
Weight-1: Weight in kilograms at cART initiation;
Weight-2: Weight in kilograms at the time of data collection
WHO: World Health Organization.
